# ‘Out of My Hands’: Palestinian Referral Care in East Jerusalem After October 7, 2023

**DOI:** 10.1111/bioe.70077

**Published:** 2025-12-26

**Authors:** Pieter Dronkers, Zeina Amro

**Affiliations:** ^1^ University of Humanistic Studies Utrecht The Netherlands; ^2^ Birzeit University Birzeit Palestine

**Keywords:** care ethics, East Jerusalem, moral distress, oncology, Palestine, referral care

## Abstract

This paper examines the moral experiences of Palestinian healthcare professionals working at a specialised referral hospital in East Jerusalem during the early months of the Gaza War. Drawing on semi‐structured interviews with hospital staff providing oncology care, it analyses how understandings of what constitutes “good” care in a context of occupation, segregation, and genocidal violence are shaped by competing normative frameworks. Three ethical perspectives initially emerged: medical ethics, which prioritises evidence‐based protocols; humanitarian ethics, which emphasises neutrality in line with donor requirements; and communitarian ethics, which understands care as an expression of solidarity and national duty. The paper argues that to fully capture the moral labour of healthcare workers, a fourth frame is needed: care ethics, which highlights the relational and reparative dimensions of care work. This perspective is crucial in a context where occupation and segregation deeply affect the “care triangle” of patients, companions, and healthcare providers. Viewing care as a relational practice that resists division and exclusion also offers a strategy to alleviate the moral distress that can arise when professionals are unable to provide care they themselves see as “good enough.” The paper calls on employers, communities, and colleagues to value and support the care work professionals provide beyond their clinical tasks, while also recognising the limits of individual responsibility by acknowledging and addressing the structural barriers they face. Ultimately, an end to occupation and segregation remains the primary condition for enabling good care.

## 1 Introduction

One of the many devastating consequences of the Israeli closure of Gaza's borders has been the trapping of patients in need of specialized referral care outside the Strip. Since October 7, 2023, Israeli crossings have remained almost hermetically sealed. In comparison, in 2021, when entry into Israel was already severely restricted, around 15,500 patients applied for permits to access treatment in Israel, East Jerusalem, the West Bank, or abroad, and about two‐thirds were approved [1]. Egypt facilitated some referrals until the Israeli capture of the Philadelphi corridor in May 2024, and restrictions briefly eased during the early 2025 ceasefire, yet by September 2025, when this paper was finalised, the World Health Organisation reported that only three patients per day could leave the Strip, while an estimated 15,000 required urgent treatment elsewhere, mainly for trauma and oncology [2]. At the same time, Gaza's healthcare infrastructure has been systematically destroyed, including its only oncology clinic, leaving many—among them around 100 cancer patients per day—without adequate care [3]. Palliative and mental healthcare are almost entirely inaccessible [2]. Overall, life expectancy in Gaza is estimated to have nearly halved since the start of the war [4].

Meanwhile, Gazan patients and their companions who were in East Jerusalem, the West Bank, or Israel on October 7, 2023 for medical treatment were unable to return home or did not dare to do so because it had become a deadly war zone. While the immediate impact of the border closure is of course most acutely felt by the patients, Palestinian referral hospitals—especially in East Jerusalem—and their staff have also been deeply affected. While improvisation and flexibility had always characterized treatment plans of these clinics, now they were not able to provide patients they had already committed to any of the treatments and drugs they needed. This not only caused a disruption of care, but also a severe decrease in turnover and a general sense of failing the patients. The war's impact on the personal lives of healthcare workers also put a burden on their professional functioning.

Although the scale of destruction in the Gaza Strip is unprecedented and the Israeli actions against the Palestinians living there qualify according to many as genocide, the effects of the border closure are related to preexisting processes of surveillance and segregation that have long shaped Palestinian life under occupation [5]. The war has once again revealed the extent to which access to healthcare in Palestine is controlled by Israel. Especially since the Second Intifada and the increased Israeli border control through the construction of the West Bank barrier, the right to healthcare has become intertwined with the freedom of movement, with both under severe strain. The increasing restrictions of these two fundamental rights affect all sides of what we will call the “care triangle” at the heart of referral healthcare: the patient, who needs specialised treatment and drugs; informal caregivers who wish to offer emotional and practical support in times of pain and distress; and healthcare professionals, who are trained to provide the best possible care. The Gaza War has further exacerbated the situation.

Under these circumstances, providing care that is “good enough” is not only a practical, but also a moral challenge. This paper examines how political and systemic impediments—compounded by the Gaza War—affect referral care in East Jerusalem, focusing on the lived experiences of healthcare professionals. Through the lens of the care triangle, the study explores how these barriers generate moral distress among healthcare workers and identifies the ethical frameworks they use to navigate these challenges. Ultimately, it advocates for *care as a relational practice* as a key framework to mitigate moral distress and sustain morally ‘good‐enough’ care in contexts of occupation and segregation.

We start by briefly highlighting (Section 2) how Palestinian referral care is specifically shaped by two characteristics: the limited freedom of movement and the fact that it to an important extent depends on humanitarian funding. We elaborate how these elements result in a specific dynamic in the “referral” care triangle of patients, companions and professionals. After that, we introduce (Section 3) the methodology of our empirical research at a Palestinian tertiary care hospital where we explored how the war put this triangle further under strain. Then, we report (Section 4) the results from our interviews with healthcare professionals. We describe their observations of how the war impacted healthcare provision. Finally, we discuss (Section 5) the different moral frameworks that professionals draw upon to determine what counts as morally “good‐enough” care in this context. We argue that approaching care as a relational practice not only highlights the specific challenges of providing care under systemic constraints but also offers a promising way to mitigate moral distress by opening moral space for professionals to reflect on and navigate questions of good care, particularly when they feel their work might fall short from the perspective of medical, humanitarian, or communitarian ethics [6].

## 2 Relationality and Referral Care

In this paper, we study care, including professional healthcare, as a fundamentally relational practice. Political scientist Joan Tronto argues that care and caring consist of several elements: recognizing and taking the responsibility for the needs of others, providing care competently, and acknowledging that, ultimately, it is the recipient who is best positioned to indicate to what extent the care provided indeed responded to their needs [7]. In healthcare, patients, professionals, and informal caregivers, such as family members, all form part of this relational web, or “care triangle.” To assess what constitutes, from an ethical perspective, as “good” or “good enough” care, it is crucial to reflect on how care can be organized in such a way that the power within these relationships is distributed fairly, preventing domination by any of the parties, including the institutional and political systems in which the care triangle is embedded. This to ensure a fair degree of agency for all involved. Such a balance is easily disrupted, for instance, when care providers undermine the autonomy of care recipients by adopting a paternalistic attitude [8]. Or, in the opposite direction, when care is primarily regarded as a commodity, available to those who can afford it, and its quality is determined solely by the level of customer satisfaction [9].

In Palestine, this web of care is under immense pressure for at least two reasons. First, one of the building blocks of the Israeli settler‐colonial system of occupation and segregation is a permit system that severely restricts the extent to which patients can physically access healthcare providers, which is crucial for proper diagnosis and treatment. The permit application process is time‐consuming, and the outcome is uncertain. From 2019 to 2021, the average approval rate for patient permit applications from Gaza was 65% [1]. The rest of the applicants was denied access, or, more often, patients had not received a decision by the time of their scheduled hospital appointments.

Not only do patients require permits to meet with their healthcare professional, but so do their family members and friends. Israel allows one, or in other cases, two companions to travel with the patient, but these individuals must undergo the same lengthy application process and security screenings. As a result, patients cannot always bring their preferred companions. With the rest of their support network left behind, the patient is often accompanied by just one person, who is expected to provide psychosocial care and acquire basic medical skills to perform certain procedures at home, as professional nurses are not always available. This situation gives the companion a unique role in the Palestinian healthcare system, which we will explore in more detail later. Ultimately, the ability of the three points of the care triangle to meet one another is entirely controlled by Israeli authorities. While international law obligates the occupying power to ensure equal access to healthcare, in practice, Israel put a restrictive regime in place, resulting in systemic discrimination, and segregated care provision. In a 2024 advisory opinion, the International Court of Justice described the restrictions on Palestinians in the West Bank as a violation of Article 3 of the International Convention on the Elimination of All Forms of Racial Discrimination, which mandates governments to prevent, prohibit, and eradicate racial segregation and apartheid [10].

The second pressure on the care triangle stems from the humanitarian funding structure of referral care. Healthcare in Palestine is partially paid by the Palestinian Authority, which is dependent on tax revenues collected—and thus controlled—by Israel. In addition to government funding, individuals also rely on private contributions, including family support and loans, to cover medical costs. However, specifically the specialised referral care in East Jerusalem is also supported by international donors who provide funding as a form of humanitarian aid. This type of financing is inherently conditional; it can be awarded or withdrawn for political or administrative reasons at any time, including as a form of collective punishment [11, 12]. As a result, the financial foundation of referral hospitals, as well as the continuity of care provision are fundamentally precarious. Both the permit system and the funding structure keeps Palestinians dependent on the benevolence of external forces.

To fully assess the impact of the political context on healthcare provision, quantitative research is key in mapping limited access and the years of life lost and the general effects it has on healthcare professionals [13]. However, exploring its impact on the care triangle through studying meanings and lived experiences in care practices through a qualitative design offers another vital perspective [14, 15]. The effects of the Israeli occupation and segregation are particularly evident in long‐term, specialized therapies where patients return repeatedly to the hospital, allowing deeper relationships to form between caregivers and patients. In such cases, the question of what constitutes as morally “good” or “good enough” care becomes especially urgent, as mutual relationships develop and caregivers become more invested in the patient's fate. This is especially true in oncology care, where difficult choices often arise, such as whether to continue treatment. For example, when the decision is made to stop a therapy, psychological support from both formal and informal caregivers becomes crucial.

It is within this care triangle that moral distress can emerge. Since the COVID‐19 crisis, academic interest in moral distress has increased significantly [16]. The term refers to the experience of recognizing the needs of others and having the will to help, but being unable to do so because of institutional or political constraints [17, 18]. The distress itself is not inherently moral, but tracing its origins serves to highlight situations where the normative standards that caregivers have conflict with the (lack of) their opportunities to act. Moral distress was first described in the context of nursing, as nurses are often the closest to patients and the first to recognize unmet caring needs [19]. However, this distress is not limited to this group, in fact all formal and informal caregivers can experience it [20]. The extent to which it is felt depends on the normative ideals individuals apply. For instance, care that falls short of medical standards may still be deemed ‘good enough’ from a humanitarian perspective.

In the rest of this paper, we explore the lived experiences of healthcare professionals providing care to cancer patients. We examine how the Gaza War, alongside preexisting systems of control and surveillance—such as occupation, segregation, and the humanitarian condition—has disrupted the care triangle and challenged the ability of hospital staff to provide care that aligns with their own normative standards, resulting in experiences of moral distress [21].

## 3 Methods

### 3.1 Research Design

This project began in September 2023 as a qualitative descriptive study of the lived experiences of healthcare professionals working in referral care. This design was selected in order to capture individual experiences of moral distress within the context of occupation and segregation, rather than to quantify its prevalence using questionnaires developed and validated for standard hospital environments [22]. The original research plan combined observations at a hospital in East Jerusalem with face‐to‐face interviews and focus groups in departments that provide oncology treatments and related services, such as palliative care and psychosocial support.

However, following the revocation of most permits by Israel after October 7, 2023, the second author, who resides in Ramallah and was responsible for fieldwork and conducting interviews in Arabic, was no longer allowed to travel to East Jerusalem. As a result, only 2 days of observations were completed prior to October 7. The data collected was limited to two introductory rounds at the hospital, accompanied by the chief nursing officer. This was used as background knowledge during the data analysis.

Despite these challenges, the decision was made to continue the research after consulting with the chief nursing officer and assessing the appropriateness of interviewing hospital staff. All interviews were from then on conducted online. Throughout the research project, the first author, who holds a European passport and was able to travel via Ben Gurion Airport in Tel Aviv, made three visits to East Jerusalem to conduct additional background interviews in English with the hospital management. During these interviews, the second author participated remotely from Ramallah.

### 3.2 Case Definition and Participant Selection

East Jerusalem has a network of six referral hospitals that serve Palestinians from the West Bank and, until October 7, 2023, also from the Gaza Strip. This article focuses on Augusta Victoria Hospital (AVH), the second largest member of this network. AVH was selected because over 90% of its patients come from the West Bank and Gaza, a higher percentage than any of the other referral hospitals. Additionally, AVH specializes in (paediatric) oncology and nephrology, areas that often require repeated visits, fostering strong relationships between healthcare professionals and patients. Due to these specializations, the hospital also has dedicated palliative care and psychosocial support departments. As AVH operates as a project of the Lutheran World Federation (LWF), the humanitarian condition elaborated in the previous section significantly influences how the hospital is positioned and perceived by both international donors and the Israeli authorities.

In coordination with AVH senior management, a sample list of staff members to interview was developed based on criteria set by the authors. These criteria included achieving a balanced representation in terms of age, career stage, and gender, which would enable us to capture how professionals with varying degrees of expertise and experience aim to care for and interact with patients and companions. This purposive sampling method was important in order to have varied experiences from the hospital staff. After the initial round of interviews, a mix of convenience and snowball sampling was employed to recruit additional respondents who met these criteria. To include a broader range of perspectives, we also interviewed one physician.

In total, eight nurses of various ranks, three psychosocial workers, and one physician were interviewed (*n* = 12). Among them, seven worked in the oncology ward, two in the palliative care unit, and three in the psychosocial department. Three staff members who were approached did not want to participate, mainly because of time constraints. Additionally, we conducted eight background interviews with members of senior management in August and November 2023, as well as January and April 2024, to understand developments at the hospital.

### 3.3 Data Collection

The data collection took place from September 2023 to January 2024. Semi‐structured interviews were conducted using a topic guide that covered various areas, including the respondents' professional careers, their tenure at AVH, their roles and involvement within their departments, their relationships and interactions with patients and companions, and memorable or particularly challenging moments of care provision where they experienced moral distress. Additionally, the practical obstacles they faced in reaching and performing their work were examined, along with their ideals about good care and their motivation to continue in their roles.

In most cases, the full topic list was addressed, though certain points took precedence depending on what participants found most pressing. After October 7, 2023, the Gaza War became a prominent theme, often taking up a significant portion of the interviews. Each interview lasted approximately 60 min. All interviews with the staff were conducted in Arabic by the second author to enable respondents to express their experiences in their first language. The interviewer has a background in and experience with qualitative and specifically ethnographic methodologies based on her own PhD research, as well as her experience as a lecturer in research methodology at Birzeit University and her previous employment as a research assistant at the Institute of Community and Public Health at that same institution.

### 3.4 Ethical Considerations

The study received approval from both the Ethical Review Committee of the University of Humanistic Studies in the Netherlands (#2023‐03) and the AVH Ethical Committee (approval dated 23 March 2023). To avoid any pressure from superiors, the nurses included in the sample were approached directly by the second author. Management was not informed of who agreed to participate.

Before deciding to take part, respondents were provided with an information sheet detailing the study's purpose, potential risks and benefits, their rights to privacy, anonymity, and confidentiality, as well as their right to withdraw at any time. At the start of each interview, an informed consent form in Arabic was presented. Participants were given time to read the information, and any necessary clarifications were provided. All participants either signed the consent form or gave their approval which was recorded digitally on an audiotape. One participant asked not to have the conversation recorded; in this case, notes were taken and included as part of the study data.

To minimize security risks, participants were identified on the audiotape or in the notes only by gender, age, and department. Given the sensitivity surrounding residency due to the Israeli permit and Jerusalem ID‐system, this information was not discussed. According to general statistics provided by AVH, 84% of staff live in the West Bank, with the percentage rising to 96% among nurses.

Immediately after the interviews, the files were securely stored on a dedicated research drive at the University of Humanistic Studies in the Netherlands. An experienced academic transcriber in Nazareth processed the de‐identified audiotapes. These audio files were deleted after the Arabic transcripts were checked for accuracy. Although individual member checks of the interview transcripts were not conducted, the authors organised a collective validation process through a roundtable discussion at the hospital with interviewees and other interested staff members. During this session in May 2024, the aggregated themes from the data analysis were shared and discussed to assess their validity and to explore their interpretation. This group feedback served as a form of member checking at the collective level. In addition, as part of the iterative validation process, a written report of the findings was shared with hospital management. Their feedback was incorporated into the interpretation of the results.

### 3.5 Data Analysis

The second author led the coding of the Arabic interview transcripts. A blended approach was used, combining both deductive and inductive coding methods [23]. This allowed the analysis to focus on pre‐defined themes, while also leaving room to explore topics and experiences that were particularly meaningful to the respondents [24]. To maintain a close connection to the project's focus on the relation between the three different positions at the care triangle, the impact of the political context, and moral distress, the coding process began with a list of sensitizing concepts tied to these themes [25]. Additional codes were introduced throughout the analysis as new topics emerged from the interviews.

The interpretation of the interview data was discussed online with the first author at various stages of the process. In January 2024, both authors held a joint session in Ramallah, during which they reviewed all the interviews together. These were translated into English on the spot, and the authors discussed the emerging themes, cross‐checking them with the topic list initially developed by the second author.

The decision to focus on a relatively small number of interviews reflects a commitment to gaining deep insight into the lived experiences of healthcare professionals. As qualitative researchers, we start from the idea of “petite generalizations,” which aim to offer an understanding of what it means to provide care in a specific political context through a thorough depiction of lived experiences [26, 27]. These detailed depictions allow readers to assess the extent to which our conclusions may be relevant to other situations.

In this paper, we balance our desire for precision with the need to protect the anonymity of respondents. Consistent with our approach of prioritizing depth over breadth, the voices of a small number of respondents will feature prominently in the following sections, as we believe these offer the most insightful perspectives on providing referral care in times of war and the moral distress it entails.

## 4 Findings

In this segment we explore how disruptions of the care triangle affect the daily work of healthcare professionals and how this can result in moral distress. We use the three points of the care triangle as the basis of our description. Each time, we start by introducing the types of disruptions, followed by a depiction of how professionals assess and respond to them.

### 4.1 Patients

Accessing specialized tertiary care requires navigating medical and bureaucratic procedures to obtain a referral, being allowed to travel to the designated hospitals, and having the means and physical condition to do so—all challenges that, for Palestinians, cannot be taken for granted. In the hospitals in the Westbank and, already before October 7, 2023, the Gaza Strip, diagnostic tools or expertise are not always available or up to standard, largely due to Israeli import and travel restrictions. So, from the outset, the accuracy of the diagnosis is uncertain and requires further verification. Then, obtaining approval and compensation from the Palestinian Authority for a referral can be a lengthy process. The Israeli permit system adds further uncertainty: patients are never sure whether they will be allowed to attend their scheduled appointments or treatment sessions. Finally, the journey to the hospital can be a challenge. Patients, often already in pain, must cross checkpoints, switch cars or ambulances, and the actual costs of this journey can be prohibitively high [28].

The challenges the patients face even before they arrive at the hospital, have a direct impact on the staff's daily work. To start with, when patients arrive for the first time, the diagnostic process must be carefully checked or even repeated. Consequently, once the diagnosis is confirmed, the head nurses are tasked with scheduling follow‐up appointments for treatment sessions, while also ensuring that the correct medication or equipment is available. If, during the course of the therapy, a patient is not allowed to attend one of the follow‐up appointments, these nurses must not only reschedule the sessions but also seek to arrange for another patient who can actually make it to the time slot that became available. Throughout this entire planning and replanning process, the nurses also have to factor in the opening hours of checkpoints, which are particularly unpredictable and restrictive since the beginning of the Gaza War. Moreover, any cancellation of a session might impact the effectiveness of the treatment plan, which then also must be revised. However, even this ingrained flexibility was insufficient to mitigate the impact of the full closure of the Gaza Strip: all therapies for Gazans had to be cancelled. “*They are out of my hands*”, as a nurse shared:

Even under normal circumstances patients from Gaza have to wait sometimes up to three to four months to be able to get to Jerusalem and access treatment, which often complicates their cases. Since the war, the restrictions increased, and patients can no longer get permits to access treatment. Any care we have provided to patients in the last few months was insufficient, especially when we are talking about chemotherapy, because the treatment journey is disrupted. *[…]* For our patients to access proper medical care, we need to eliminate the checkpoints!

In October 2023 alone, at least 39 Gazan patients missed their radiation sessions, and up to 180 others were unable to receive chemotherapy [29]. The management reports a 35% drop in surgeries performed. A physician we interviewed stated:

60% of our AVH child patients are from Gaza. *[…]* We had hoped, wished, to get them all here. I wish there was a way to coordinate that they could come here, but that was completely impossible. Not only that but some of the patients who were already in Jerusalem were returned *[by Israeli forces]* to Gaza. Did you hear the story when they raided the Al Makassed hospital? Right after the start of the war, they *[the Israeli forces]* came and took patients and companions from the hospital and sent them to Gaza *[* 30 *]*. There are red lines which should be forbidden to be crossed, which are now really being crossed. We are seeing things we've never seen.

In the first months of the war, the hospital explored various options to deliver medicine and supplies to patients in Gaza, which would have allowed them to perform some medical procedures themselves. However, all efforts by the hospital management, as well as by individual staff members were unsuccessful. One nephrology nurse shared:

How are we supposed to provide them *[patients]* with the materials so they would have their dialysis? One patient *[from Gaza]* even went on the radio and was interviewed about her situation. She said she only has one week left before all of her treatment supplies would be finished. *[…]* We were unable to provide them and couldn't stay in communication with her. Since the war the hospital has not been able to serve our patients in Gaza anymore.

Despite the challenges, staff members went at great lengths to remain in contact and check on the well‐being of their patients. Especially, when nurses had strong bonds with a patient, they felt a sense of duty to provide emotional support in the absence of other forms of care. One nurse spoke about a patient who had become especially dear to him:

We're of the same age, so I don't know really, but maybe the friendship just developed naturally and emotionally. This patient from Gaza has been getting treatment for around four years here. I find myself constantly wanting to reach out, wanting to ask how things are now in Gaza, how they're doing. I even wonder if he was martyred, God forbid. I want to know how things are for him.

Another nurse shared how much the situation of their patients affected her:

I asked from a place of worry how he's doing. He *[the patient]* responded that things are difficult: ‘Just pray for us.’ After that *[contact]* no longer asked and didn't hear from him anymore.

For the approximately 50 patients from Gaza who were at the hospital on October 7, 2023, returning home or not became an impossible choice. While staying in Jerusalem for their treatment, they lived in constant anxiety, fearing for their loved ones. Some patients from Gaza have even considered giving up on their treatment or refused to eat, feeling that continuing it served no purpose. A social worker shared:

There was one young woman who was here, a mother whose child was in Gaza and she was here at the hospital. Her husband used to work in Israel. It was very difficult for her. She was constantly crying. I was afraid that her *[kidney]* transplantation would fail, because her psychological state was bad, very bad. So you always had to be with them, you had to support them, calm them down a little bit from their fear, from their tensions, from their anxiety due to the war.

While travel with a medical permit from the West Bank to the hospital remained possible for most of the war, the security situation has made it too risky for the palliative care unit to visit patients in their homes. Since there is no community nurse network in Palestinian villages, palliative nurses had recently started visiting West Bank patients after discharge to ensure adequate pain relief. This also became an important way to free up hospital beds for new patients. When these home consultations were no longer possible, it affected both the scope and the quality of palliative care, as one nurse noted:

Many of the patients now have to go back to the hospital instead of receiving home visits. They were happy with the comfort these visits provided, but this is no longer possible. I mean, we used to relieve the patients of the huge burdens of coming to the hospital. I mean, you know, usually they'll have to use ambulances, to coordinate, to pay huge fees. Not to mention the exhausting experience itself.

The quotes highlight the numerous ways in which occupation and segregation complicate and even impede care provision, requiring healthcare professionals to invest significant extra time and energy in their work. They adapt treatment plans, provide psychosocial support during difficult moments, and strive to remain in communication with patients, even when it is uncertain if they will ever see those patients again. At the institutional level, the hospital also takes measures to mitigate disruptions in the care triangle: it employs staff to assist with the permit process, operates bus services to ease checkpoint crossings, and offers psychosocial support that extends to practical needs like housing and food, particularly for patients from Gaza who need to stay in East Jerusalem. Despite these efforts, the quotes above also reflect the frustration, anger, anxiety, and disappointment that healthcare professionals endure when they are unable to deliver care that they believe to be good or at least good enough.

### 4.2 Companions

Companions play a unique role in Palestinian referral care. For patients, they are the only member of their social network they can directly rely on for mental and practical support. Companions are also key figures for healthcare professionals. Especially since communication networks in Palestine are not always reliable, companions are often the first point of contact when medical decisions need to be made, particularly when patients are minors or are in a very weak condition [1]. As one respondent phrased it, companions are “*the eyes of the nurse on the ward.*” Staff members expect these informal caregivers to be fully committed to the patient and present on the ward at all times. Additionally, during the hospital stay, nurses train companions in performing basic medical procedures and in recognizing and reporting symptoms or potential relapses after discharge.

A first disruption at this side of the care triangle occurs when patients cannot bring their preferred companion. Ultimately, it is the Israeli authorities who determine which companion, if any, receives permission to accompany the patient. In many cases, only an older relative, a more distant family member or even someone from the patient's broader social network is granted a permit [1]. The fact that companions are often not first‐degree family members presents dilemmas for healthcare professionals regarding the amount of medical information they can share. Respondents explained that while they strive to uphold patient privacy, withholding information from these more distant companions can lead to feelings of exclusion, potentially reducing their engagement and reliability in supporting the patient's care.

Another disruption happens when companions leave the hospital compound to make use of the rare opportunity to visit the old city of Jerusalem and especially its holy places. One respondent recounted such an incident with lingering indignation:

For example, a mother goes out at night or goes, for example, on Friday to the Al‐Aqsa Mosque. She leaves her child at the hospital and just goes. This is definitely not permitted, and it should not happen because they are under the responsibility of the hospital. How could she just leave him and go?! And this also creates problems for the nurses and for nursing.

For companions, managing the high expectations and all the different tasks is challenging, as they also have to cope with their own emotions while being isolated from their social support networks and unable to fulfil other responsibilities they have at home. Therefore, unlike other contexts, healthcare professionals at AVH must dedicate time not only to patients but also to companions to ensure they can fulfil the roles expected of them. For instance, when patients from Gaza became stranded in Jerusalem, the staff members also needed to address the needs of around 50 companions who travelled with them. A social worker describes:

We worked with them through individual and group sessions, where they would talk and share about the situation their families are living in in Gaza, and about their feelings here *[in Jerusalem]*. At first, they felt a lot of guilt, that they are safe here, and their children are not safe at all. They felt guilty that they have food provided, and some children may even not like it, but they know that in Gaza people can't find anything to eat.

Next to psychosocial care, the hospital also had to organize practical support, including accommodation, clothing and even medical aid. The social worker continues:

Many of the companions, especially the older ones, have chronic illnesses, and had medication for only a certain period, which soon ran out. So the hospital set a policy of opening a clinic twice a week for the companions. They get checked by doctors. They have their medications biweekly. The hospital has spared nothing: I believe it has done its duty and more.

In Palestine, the figure of the companion embodies the limitations to the freedom of movement. Given their crucial role in the care triangle, the companions' absence or reduced involvement immediately shifts additional responsibilities to healthcare professionals. Moreover, companions themselves require care, further increasing the professionals' workload. These factors are potential sources of moral distress, as healthcare professionals may feel they must compensate for gaps left by the companion, or assume responsibility for an informal care provider whose vital role is neither institutionalized nor officially recognized.

### 4.3 Healthcare Professionals

Disruptions affecting the professional side of the care triangle are also closely tied to Israeli movement restrictions, as they have a direct impact on the many employees commuting from the West Bank. Like their patients, hospital employees are dependent on acquiring and keeping an Israeli travel permit, which involves, for instance, sacrificing privacy and the risk of being pressured to share information or to even become an informant [31]. Then there is the daily commute to the hospital, which means starting the workday by spending far more time on transportation than the actual distance requires. The challenge is more than logistical; it is also mentally taxing to endure long waits at checkpoints, undergo screening by Israeli soldiers, and, if questioned, suppress any reaction that could lead to further delays. By the time employees arrive at the hospital, they may already be fatigued or processing the stress of checkpoint experiences, yet they must quickly shift focus to attend to their patients' needs.

Dealing with these limitations to freedom of movement has become a routine aspect of life under occupation. After October 7, 2023, however, the emotional strain intensified. As one nurse explained:

I have to cross several checkpoints to travel from the northern West Bank to Jerusalem. To be honest, in general, that is not too difficult, but it nevertheless entails many challenges. Now, however, we have to cross checkpoints in an abnormal context. Now we are scared, but we still want to get to work.

Despite these difficulties, respondents from the West Bank shared that AVH remains an attractive employer. First, as the main clinic for oncology and nephrology care, AVH provides an engaging workplace for those trained in these specializations and who look for a professional challenge. Second, respondents expressed a commitment to using their expertise to benefit the Palestinian community, even though their skills could secure positions elsewhere, such as in the Gulf States. They consider their work a duty to serve fellow Palestinians. Third, the fact that salaries in East Jerusalem are higher than in the West Bank—though still lower than in Israeli hospitals—adds to AVH's appeal, especially since the war's start, which has led to staggering unemployment and poverty rates in the West Bank [32]. Although AVH introduced temporary 30% salary retentions for a limited number of months, employees were eventually compensated. As a nurse noted:

Right now, you cannot sacrifice a guaranteed job. You take into consideration that many are unemployed, while you have work. So you thank God that you have a job. For example, in some other institutions they are only paying half a salary to their employees, and I also heard stories of others who are going several months without pay, so you come to the conclusion that it is better to remain where you have job security.

Even if people had wanted to change jobs, they did not have the luxury to do so. Moreover, AVH introduced mitigating measures to address the risks of commuting. Next to the already existing collective transportation from major cities in the West Bank, it provided accommodation on its compound for those who wished to work several shifts in a row to reduce the number of commutes, or who opted to travel home only once a week for the weekend. This was especially important at the beginning of the war, when tensions were high and checkpoints often closed. AVH also adjusted work schedules, allowing nurses flexibility to switch shifts with one another, thereby facilitating the mutual support colleagues were prepared to offer each other.

This readiness to step in for one another demonstrates the sense of solidarity that emerged at the beginning of the war and that healthcare professionals described in the interviews. Staff members from the same areas began coordinating to travel together. Carpooling was a way to increase personal security and reduce stress and anxiety. A nurse explains:

You know, between Qalqilya and the Qalandia: we are talking about almost 12 settlements, all this in addition to the checkpoints: the road is not easy, and it is completely different from what it used to be the past 12 years.

Due to the unpredictability of the situation, employees shared that they felt forced to leave their homes even earlier than before the war just to make it to work on time. A nurse summarises:

Our shifts had to be organised according to what region we are from and whether we can get to the hospital. Today, for instance, I am here two hours early because you cannot guarantee that you can arrive on time. Still, there is a sense of understanding between everyone. For example, if there are difficulties on the roads for me or my colleague, someone can cover for us. There is agreement on this.

The extended commute further reduced the time employees could dedicate to personal life and family duties. An increased sense of fear and anxiety became the norm. However, during a roundtable discussion on our findings, several participants shared that their greatest source of moral distress was the fate of their colleagues and patients in the Gaza Strip. Witnessing hospitals being attacked and destroyed, while fellow professionals continued to provide basic medical care, was deeply distressing and terrifying, as healthcare institutions had apparently become deliberate targets of war.

The distress ran even deeper because AVH had been on the verge of opening a diagnostic facility at Al‐Ahli Arab Hospital in Gaza City. Local staff had already been hired and had completed their final technical training session at AVH in September 2023. Yet, already in the first weeks of the war, Al‐Ahli sustained severe damage, preventing the new clinic from operating [33]. Staff members were forced to flee multiple times, though the hospital maintained contact with them and continued to pay their salaries. A €4‐million investment seemed to go to waste.

## 5 Discussion

Our findings underscore the immense challenges facing Palestinian referral care, which existed prior to but have been aggravated since October 7, 2023. Patients struggle to access specialized care due to movement barriers and treatment disruptions. Companions—vital sources of emotional and practical support—are often prevented from fulfilling their roles because of permit restrictions. Healthcare workers face daily obstacles, from exhaustingly long commutes to mounting anxieties, while adapting their work routines to an ever‐changing landscape of occupation and violence. Together, these conditions not only impede the delivery of good care but also generate significant moral distress for professionals who find themselves unable to uphold their ideals and values.

What qualifies as “good care” in this context depends on the professional standards or normative frameworks healthcare providers, their employers and their patients adopt. In this section, we first examine the guidance offered by medical, humanitarian, and communitarian ethics, and then demonstrate how care ethics can integrate these three approaches, addressing their gaps and limitations in a cohesive and productive way.

The dominant framework emerging from the findings is that of medical ethics, which includes principles such as autonomy, non‐maleficence and autonomy. Like any other hospital, AVH is committed to delivering the highest standard of medical care, prioritizing adherence to well‐established protocols and evidence‐based treatment plans to ensure optimal outcomes. Its quality assurance system underpins AVH's accreditation by the Joint Commission International, qualifying it as one of the Palestinian Authority's main contractors for specialized oncology care [34]. Within this normative framework, adapting treatment plans for non‐medical reasons or having to halt treatment entirely exemplifies not‐good‐enough care. The interviews also revealed more mundane breaches of medical ethical standards, such as sharing confidential information with companions to ensure engagement or making urgent treatment decisions without consulting a patient's family back home due to the obstacles impeding communication. While such actions may violate the principles of autonomy and privacy, they can still be justified in this context as ultimately benefiting the patient.

The second framework is that of humanitarian ethics, which includes principles like neutrality and independence. Adherence to humanitarian neutrality forms the basis of third‐party funding and provides some protection from direct Israeli interference on the compound. Therefore, providing good care requires at least a partial alignment with humanitarian principles to secure international support. Accordingly, while AVH emphasizes that international funding should be accompanied by political support, it primarily frames this solidarity in humanitarian terms [35]. This approach is reflected in the hospital's visual environment and its educational programme: the international logo of the Lutheran World Federation (LWF) is visible throughout the compound, there are no overt symbols of Palestinian nationhood, and employees receive training in the LWF's international code of conduct [36]. In fact, during the roundtable we organized to present our research findings, some staff members noted that it was one of the first times they openly discussed how the broader political context affects their work. However, even the current level of humanitarian neutrality the hospital feels compelled to maintain does not guarantee stable operations. At the start of the Gaza War, the European Union and other donors threatened to suspend all funding to Palestinian institutions [37]. Had these threats been carried out, the referral hospitals in East Jerusalem would have faced severe financial difficulties. Layoffs due to funding cuts would have further deepened the impoverishment of the Palestinian population in the West Bank, underscoring the precarious nature of healthcare provision under humanitarian conditions. As long as international donors fail to recognize and openly address the root causes of the current fragmented care provision, Palestinian healthcare will remain conditional, precarious, and dependent on foreign aid [38].

The third framework surfacing in the interviews is that of Palestinian identity and communitarian ethics. Employees view their work as a duty to support fellow Palestinians with life‐threatening diseases who live in dire circumstances. Their loyalty to their patients persists, even in the most challenging circumstances. Paradoxically, while AVH tries to maintain a high level of humanitarian neutrality, it is also a very noticeable symbol of Palestinian presence in East Jerusalem, where few functioning Palestinian institutions remain [39]. This symbolic significance extends beyond its striking building on the Mount of Olives with its high church tower: Palestinian Jerusalemites volunteer at the hospital to visit lonely patients, students from Palestinian high schools get tours and do small assignments at the compound, and during Ramadan, Jerusalem Muslims bring gifts for the patients. In 2019, when AVH encountered a financial crisis due to delayed reimbursements for treated patients, the Palestinian community in Jerusalem organized a successful fundraising campaign, underscoring the hospital's symbolic importance as a site of solidarity and national identity [40]. Within this moral framework, good care is an expression of solidarity with fellow Palestinians—even if it is as simple as nurses maintaining contact with those who can no longer access the hospital.

In the first sections of this paper, we introduced a fourth normative framework: care as a relational practice. This perspective recognizes dependency as a fundamental human condition and stresses the importance of weaving and repairing a life‐sustaining web that supports human flourishing. Although this approach is especially well developed in nursing ethics, it applies to all healthcare professionals and is particularly relevant in a context where freedom of movement is severely restricted, and patients' support networks are under constant strain [41]. By centring on relationality, this framework highlights that much of the care work underlying the other three normative frameworks involves maintaining and restoring connections. The interviews show the time and effort professionals invest in the repair work needed to sustain the three sides of the care triangle: the patient, their network through the companion, and the healthcare workers and their colleagues. From this viewpoint, good care means recognizing the gaps in this triangular web and taking responsibility to mend them wherever possible. On the one hand, a care ethics perspective focuses attention on the everyday practices of rebuilding damaged connections at the bedside of the patient. On the other hand, it also brings the political dimension of care work into view. In Palestine, the fragmentation of these connections is largely a result of Israeli actions. Thus, discussing what constitutes good care is inseparable from critically analysing the political context that shapes it [42].

Across these four normative frameworks we have presented, care can be simultaneously good and not‐good‐enough, depending on the moral perspective applied. To relieve moral distress, it is crucial to draw on all four perspectives when discussing what good care for a particular patient entails. For instance, breaching privacy protocols to maintain the engagement of a distantly related companion may be problematic from a medical ethics standpoint but justifiable within a care ethics framework. Even when providing good medical care is impossible, care as a practice of restoring relationality can continue and should be valued by all involved, including the employer. It is part of a healthcare professional's role to make these ethical judgements, be prepared to justify them, and remain accountable. It is up to the healthcare institution to provide the moral space to its employees to discuss their decisions and, if needed, to revise them [6]. Weaving human connections in a context designed to enforce fragmentation and segregation is, in itself, a subversive counter‐conduct and contestation of oppressive power structures—and thus an act of good care from a relational perspective [43].

Recognizing this oppressive context is also essential to avoid placing moral blame on the wrong shoulders. Many instances of not‐good‐enough care are directly linked to Israeli policies. For example, failing to provide patients with their medications or employees arriving late due to unexpected delays at checkpoints should not be judged without taking the root causes into account. If these structural factors are not acknowledged, there is an increased risk of exacerbating feelings of guilt and contributing to moral distress among staff members.

## 6 Conclusions

The Gaza War has had a devastating impact on Palestinian healthcare, including the referral hospitals in East Jerusalem. This paper has shown how all three sides of the care triangle—patients, companions, and healthcare professionals—have been affected. The interviews we conducted highlight how healthcare providers strive to deliver care that is “as good as possible” under extraordinarily difficult conditions.

Our analysis reveals that what constitutes good care can be understood in various ways, depending on the ethical framework employed. We argue that focusing on the relational dimension of care is especially important. This perspective not only brings to light the tremendous efforts healthcare providers invest in rebuilding and maintaining connections but also clarifies why these efforts have moral weight, particularly when other forms of care are out of reach.

When healthcare professionals cannot meet their own standards, moral distress can arise. Yet recognizing the significance of relational care can help ease these feelings of failure. In Palestine, the act of restoring connections can even be one of the most ethically meaningful responses to Israeli policies of segregation. Finally, institutional support for psychosocial care and opportunities for healthcare professionals to reflect on their experiences—both together and individually—can further sustain them in their daily work.

### 6.1 Limitations

The Gaza War significantly constrained our research. The planned fieldwork could not be fully implemented, and all interviews had to be conducted online. In a context where personal contact is essential, particularly for recruiting respondents, this presented a notable challenge. Although this study was designed as a qualitative investigation with a small sample size, we were unable to recruit the intended number of respondents or organize the focus groups as originally planned. Despite these limitations, we believe the findings presented here provide valuable insights into the challenges faced by healthcare professionals in referral care during the first months of the Gaza War.

## Conflicts of Interest

The authors report receiving a grant from the Dutch Research Council NWO during the conduct of the study.
